# A Case of Thrombotic Microangiopathy Secondary to Hypertensive Emergency: Presentation, Management, and Distinguishing Features

**DOI:** 10.7759/cureus.74498

**Published:** 2024-11-26

**Authors:** Saad Rashid, Sultan Ahmed, Mohammed Ahmed Khan

**Affiliations:** 1 Department of Internal Medicine, Mercyhealth Graduate Medical Education (GME) Consortium, Rockford, USA; 2 College of Medicine, Allegheny Health Network, Erie, USA; 3 College of Medicine, Lake Erie College of Osteopathic Medicine, Erie, USA; 4 Department of Medicine, Mercyhealth Graduate Medical Education (GME) Consortium, Rockford, USA

**Keywords:** acute kidney injury, anemia, hypertension, hypertensive emergency, thrombocytopenia

## Abstract

Thrombotic microangiopathies (TMA) are a group of conditions that present with varying degrees of microthrombi, thrombocytopenia, microangiopathic hemolytic anemia, renal dysfunction, and neurological impairment. Etiologies can be primary, such as thrombotic thrombocytopenic purpura (TTP), hemolytic uremic syndrome (HUS), and atypical hemolytic uremic syndrome (aHUS), or secondary, such as due to systemic infections, malignancies, immune-mediated conditions, and hypertensive emergencies. In hypertensive emergencies, this presentation can occur from mechanical stress placed on red blood cells as they pass through narrowed arteries due to edema and microangiopathic changes within the vessels themselves. In TMA secondary to hypertensive emergencies (HTN-TMA), blood pressure control alone can lead to improvement in cytopenias.

We present a case of a 48-year-old male with HTN-TMA. The patient had normalization of thrombocytopenia and improvement in anemia with adequate blood pressure control. This case highlights the difficulty in making this diagnosis due to overlapping presentations with primary thrombotic microangiopathies and the extensive etiologies that should be considered as part of a differential diagnosis.

## Introduction

Thrombotic microangiopathies (TMA) are a group of disorders characterized by thrombocytopenia, microthrombi, and microangiopathic hemolytic anemia [1]. Microangiopathic hemolytic anemias can be defined as primary or secondary. Common forms of primary microangiopathic hemolytic anemias include thrombotic thrombocytopenic purpura (TTP), hemolytic uremic syndrome (HUS), and atypical hemolytic uremic syndrome (aHUS). Secondary causes include autoimmune diseases, infections, and hypertensive emergencies, among others [2]. Different etiologies of microangiopathic hemolytic anemias vary in the treatment recommendations and their disease course. In the case of thrombotic microangiopathies secondary to hypertensive emergency (HTN-TMA), treating the underlying hypertension can lead to improvement in the patients’ cytopenias. Patients may require renal replacement therapy depending on the severity of the acute kidney injury (AKI), which may progress to end-stage renal disease.

## Case presentation

A 48-year-old male with a past medical history of poorly controlled hypertension (not taking any anti-hypertensive therapy), pre-diabetes mellitus (last hemoglobin A1c of 6%), chronic kidney disease stage 2 (baseline creatinine of 1.1, glomerular filtration rate (GFR) of 65 mL/min/1.73 m^2^), presented to the emergency department with one week of gross hematuria, nausea, and vomiting. On presentation, the patient was hypertensive with BP 266/163, with a heart rate of 79, and oxygen saturation >95% on room air. In the emergency department, the patient received intravenous hydralazine 10 mg for blood pressure control.

Initial labs were notable for acute kidney injury, anemia, and thrombocytopenia, with creatinine of 7.6, hemoglobin 10.4, and platelet count of 37,000 (prior outpatient labs one year prior had shown hemoglobin of 13.8 and platelet count of 214,000). Urinalysis was notable for blood, >500 protein, and >100 red blood cells.

Computed tomography (CT) of the abdomen/pelvis with IV contrast was obtained without acute abnormalities. The patient was admitted to the medical floor, and nephrology and hematology were consulted. Per nephrology recommendations, the patient underwent a thorough work-up for acute kidney injury.

Negative work-up for infectious causes of kidney injury included negative Hepatitis B and C serologies and negative HIV testing. Serologies to assess for vasculitis included negative anti-myeloperoxidase (MPO), anti-proteinase 3 (PR3), anti-glomerular basement membrane (GBM), and antistreptolysin antibodies. Complement testing (C3 and C4), angiotensin-converting enzyme (ACE), urine metanephrines, and renin-aldosterone ratio (2.4) were also all within the normal range. Electrophoresis did not show an M-spike. Kappa and Lambda ratios were elevated but with normal Kappa/Lambda ratios, thought to be secondary to kidney dysfunction.

Evidence of hemolysis was noted on lab work, with elevated lactate dehydrogenase (LDH), low haptoglobin, and elevated reticulocyte count. Peripheral smear revealed polychromasia and schistocytes. PLASMIC scoring, as part of the evaluation for TTP, was 4, and thus, the ADAMTS13 antibody was obtained. Tables 1, 2 show a thorough set of labs obtained during the inpatient stay.

**Table 1:** Select lab work during the inpatient stay. INR: international normalized ratio, LDH: lactate dehydrogenase, GFR: glomerular filtration rate.

Parameter	Patient value	Reference range
White blood cell count	6.5 x 10^3^/µL	3.6–12.9 x 10^3^/µL
Red blood cell count	3.67 x 10^6^/µL	3.9–5.6 x 10^6^/µL
Hemoglobin	10.4 g/dL	12.8–16.9 g/dL
Mean corpuscular volume	84.5 fL	80–100 fL
Hematocrit	31%	38%-50%
Platelet count	37 x 10^3^/µL	130-370 x 10^3^/µL
Prothrombin time	10.5 seconds	9.5–11.5 seconds
INR	1.0 seconds	0.8–1.2 seconds
LDH	1344 U/L	135-250 U/L
Fibrinogen	407 mg/dL	205.0-522.0 mg/dL
Haptoglobin	<10 mg/dL	30-200 mg/dL
Total bilirubin	0.9 mg/dL	0.1-1.2 mg/dL
Ferritin	902.2 ng/mL	30.0–400.0 ng/mL
Reticulocyte count	3.0%	0.61–2.62%
Immature reticulocyte fraction	4.0%	2.3–13.4%
Direct Coombs' testing	Negative	Negative
Blood urea nitrogen	66 mg/dL	6–20 mg/dL
Creatinine	7.6 mg/dL	0.7–1.2 mg/dL
GFR	8 mL/min/1.72 m^2^	≥60 mL/min/1.72 m^2^
Urinalysis	+Large blood, >500 protein, +100 red blood cells	Blood: negative, protein: negative-trace, red blood cells: none, 0-2/HPF

**Table 2:** Further work-up during the inpatient stay. anti-MPO: anti-myeloperoxidase, anti-PR3: anti-proteinase 3.

Parameter	Patient value	Reference range
Anti-MPO antibodies	<0.2 units	0.0–0.9 units
Anti-PR3 antibodies	<0.2 units	0.0–0.9 units
Antinuclear antibodies	Negative (<1:80)	Negative (<1:80)
Antistreptolysin O antibodies	57.9 IU/mL	0.0–200.0 IU/mL
Anti-glomerular basement membrane antibodies	<0.2 units	0.0–0.9 units
C3 complement	123 mg/dL	82–167 mg/dL
C4 complement	38 mg/dL	12–38 mg/dL
Serum angiotensin-converting enzyme	32 U/L	14–82 U/L
Hepatitis C antibody	Non-reactive	Non-reactive
Hepatitis B serologies	Hepatitis B surface antigen: negative, Hepatitis B core antibody: negative, Qualitative Hepatitis B surface antigen: non-reactive	Negative/non-reactive
HIV P24 antigen/HIV 1,2 antibody	Non-reactive	Non-reactive
Scleroderma SCL-70 antibody	<0.2 AI	0.0–0.9 AI
Anti-centromere antibody	<0.2 AI	0.0–0.9 AI
Thyroid-stimulating hormone	1.4 mU/mL	0.27–4.20 mU/mL
Fractionated plasma metanephrines	204.1 pg/mL	0.0–218.9 pg/mL
Renin aldosterone ratio	2.4 ng/mL/hour	0.0–30.0 ng/mL/hour
Erythropoietin	4.9 mU/mL	2.6–18.5 mU/mL
ADAMTS13 activity	100%	>66.8%

A kidney biopsy was performed, which was notable for thrombotic microangiopathy. 11/36 glomeruli were globally sclerotic. Multiple glomeruli were noted to have fibrinoid necrosis. Forty percent of the renal cortex was notable for interstitial fibrosis and tubular atrophy. Per pathology, the findings were suggestive of accelerated hypertension leading to renal damage.

Across the patient’s seven-day hospital stay, focus was placed on normalizing blood pressure with continuous nicardipine infusion on presentation and transition to oral anti-hypertensives of nifedipine, hydralazine, and carvedilol by discharge. Blood pressure on the day of discharge was 139/81. During this time, the patient’s thrombocytopenia normalized (with a platelet count of 218,000), and anemia improved (with repeat hemoglobin of 12.0 by the day of discharge).

Despite adequate blood pressure control, the patient's kidney function continued to worsen during the inpatient stay, and the patient continued to be oliguric. He was initiated on hemodialysis, which was continued through the day of discharge. The patient was discharged to follow up with nephrology outpatient with a focus on good blood pressure control and continued outpatient hemodialysis.

## Discussion

Microangiopathic hemolytic anemias can be classified as primary or secondary. Both primary and secondary forms lead to red blood cell destruction and thrombocytopenia because of platelet activation and consumption. 

TTP is caused by a deficiency of the enzyme ADAMTS13 due to genetic mutations or acquired autoantibodies. ADAMTS13 cleaves von Willebrand factor (vWf) into smaller subunits in normal function. Without ADAMTS13 function, vWf multimers form in the plasma, bind to platelets, form platelet-rich fibrin strands, and lead to intravascular hemolysis and tissue injury [3]. HUS, on the other hand, generally occurs following an infection by a strain of *E. coli *O157:H7. Infection leads to dysregulation of the alternative complement pathway, leading to increased complement activity [4]. Atypical HUS is defined by pathogenic mutations in complement genes, such as complement factor H, membrane cofactor protein (MCP), and complement C3. The disease course is complicated by relapses and can be triggered by respiratory or pulmonary infections, among other causes [5].

Hypertensive emergencies are characterized by severe hypertension and acute end-organ damage involving organs such as the brain, kidneys, and heart, among others. In rare cases, patients may develop thrombotic microangiopathy. This may be due to mechanical stress placed on red blood cells as they pass through narrowed arteries due to edema or fibrinoid necrosis due to severe hypertension [6]. Vascular endothelial cell dysfunction occurs from renin-angiotensin-aldosterone system (RAAS) activation, leading to vasoconstriction. Prolonged hypertension leads to proinflammatory cytokine release. Thrombotic microangiopathy secondary to hypertensive emergency (HTN-TMA) is associated with small vessel thrombosis, platelet consumption, intravascular hemolysis, and acute kidney injury [7]. 

It may be difficult to distinguish HTN-TMA from TTP, HUS, and atypical HUS due to an overlapping presentation. It is important, however, as treatment differs. Patient presentation and lab serologies can help differentiate between different types of thrombotic microangiopathies. A thorough evaluation of the patient’s presenting symptoms (such as diarrhea) and vitals (such as significant hypertension) and reviewing a full list of prior medications, as part of a thorough history and physical, is important. All types of thrombotic microangiopathies generally present with hemolytic anemia (high LDH, low haptoglobin, low hemoglobin, presence of schistocytes on peripheral smear), thrombocytopenia, and acute kidney injury. The severity of thrombocytopenia is often more severe in TTP as compared to HTN-TMA, and the severity of AKI is often less severe in TTP as compared to HTN-TMA, per a cross-sectional study performed in the French TMA Center comprising a panel of 214 patients [8]. In HTN-TMA, ADAMTS-13 levels are generally >20%, as compared to <10% in TTP. HUS can be diagnosed by PCR or culture-based assays for the Shiga-toxin-producing *E. coli*. C3 complement deficiency can be supportive for AHUS diagnosis. A kidney biopsy can be beneficial, as it can identify thrombotic microangiopathy as opposed to other causes of renal impairment. However, kidney biopsy generally does not differentiate between types of thrombotic microangiopathy, as typical histologic findings of arteriolar or capillary thickening, endothelial edema or detachment, and fibrin or platelet-rich thrombi are nonspecific [9].

In TTP, patients benefit from plasmapheresis, as it works to remove autoantibodies to ADAMTS13. If AHUS is suspected, plasma exchange can be started until a clear diagnosis is made. Eculizumab is a humanized chimeric monoclonal antibody directed against complement component C5, which works to block the assembly of C5b-C9, thereby preventing endothelial injury. This monoclonal antibody is used to treat atypical HUS. A longer-acting form of the antibody, ravulizumab, is also available. Currently, there are unclear guidelines regarding the optimal duration of therapy [10]. 

The PLASMIC Scoring System is one way to stratify patients into low, intermediate, and high-risk categories, who may benefit from early initiation of plasma exchange while awaiting ADAMTS13 results. Our patient had a PLASMIC score of 4, placing the patient in the low-risk category, and thus, plasmapheresis was not started. If clinical suspicion for TTP is high, with higher PLASMIC scoring, plasmapheresis should be initiated due to the mortality associated with this condition, which is >50% [11]. In our case, our patient did not present with bloody diarrhea, making HUS less likely as the diagnosis and complement C3 levels were normal, making AHUS less likely as the diagnosis.

For thrombotic microangiopathy secondary to the hypertensive emergency, neither plasmapheresis nor eculizumab are indicated. Plasmapheresis does not have any proven therapeutic benefit. It is expensive and has many side effects, including allergic reactions and increased risk of bloodstream infections.

Management of HTN-TMA involves aggressive correcting of the underlying hypertension and supportive management. Per American Heart Association guidelines for a hypertensive emergency, a maximum blood pressure reduction of 25% in the first hour, followed by a target of 160/100-110 mmHg over the next two to six hours, and then normalizing over the next 24-48 hours is recommended, outside of etiologies such as aortic dissection and pheochromocytoma [12]. Faster reduction can increase the risk of further ischemic damage. In many patients, blood pressure reduction can also lead to restoration of kidney function. Despite blood pressure correction, some patients may require renal replacement therapy [13]. Evaluation for secondary causes of hypertension should be considered. As in our case presented above, with improved blood pressure control in patient, the patient’s thrombocytopenia and anemia improved with anti-hypertensive therapy, but renal function did not. 

## Conclusions

Thrombotic microangiopathies are a group of disorders, all characterized by thrombocytopenia, microthrombi, and microangiopathic hemolytic anemia. Differentiating between primary and secondary causes may be difficult due to nonspecific symptom presentation and similar lab presentation, but it is essential in determining therapeutic options for benefit. If significant concern for TTP is present, plasmapheresis is recommended, due to high mortality from the condition. If uncontrolled hypertension is the cause, treating the underlying hypertension can lead to improvements in blood counts and kidney function.
